# Hepatocellular carcinoma and death and transplantation in chronic hepatitis B treated with entecavir or tenofovir disoproxil fumarate

**DOI:** 10.1038/s41598-020-70433-z

**Published:** 2020-08-11

**Authors:** Yeonjung Ha, Young Eun Chon, Mi Na Kim, Joo Ho Lee, Seong Gyu Hwang

**Affiliations:** Department of Gastroenterology, CHA Bundang Medical Center, CHA University, 59 Yatap-ro, Bundang-gu, Seongnam-si, Gyeonggi-do 13496 South Korea

**Keywords:** Hepatitis, Liver cancer, Liver cirrhosis, Liver fibrosis

## Abstract

Conflicting results have been reported regarding which of entecavir (ETV) or tenofovir disoproxil fumarate (TDF) is associated with better outcomes. Chronic hepatitis B patients who started ETV or TDF between 2010 and 2015 were analysed. The primary outcomes were hepatocellular carcinoma and death and transplantation. The impact of the treatment on the primary outcomes was analysed using Cox proportional hazards models in the entire and propensity score-matched cohorts. A total of 404 patients (180 and 224 in the ETV and TDF groups, respectively) were analysed. The median duration of follow-up was significantly longer in the ETV group (64.0 vs. 49.1 months; *P* < 0.001). Virological response (79.4% vs. 68.4%; *P* = 0.018) and sustained virological suppression (59.7% vs. 45.2%; *P* = 0.005) were significantly higher in the TDF group. TDF was associated with lower hepatocellular carcinoma [hazard ratio (HR) 0.31, 95% confidence interval (95% CI), 0.12‒0.79; *P* = 0.014]; however, statistical significance was not reached after adjusting sustained virological suppression using propensity score matching (HR 0.36, 95% CI 0.12‒1.14; *P* = 0.08). Death and transplantation was comparable. In conclusion, the impact of TDF on the lower hepatocellular carcinoma was blunted after adjusting sustained virological suppression. Further comparison in a larger number of patients who show sustained virological suppression over a longer period of time is needed.

## Introduction

Studies demonstrating long-term outcomes following tenofovir disoproxil fumarate (TDF) treatment have been recently reported^1–4^. A Korean nationwide cohort study identified that TDF, compared to entecavir (ETV), was associated with a lower incidence of hepatocellular carcinoma (HCC)^1^. However, a subsequent Korean multicentre study contrastively reported no difference in HCC between the two nucleos(t)ide analogues (NAs)^2^.

Virological suppression reduces hepatic inflammation, progression to fibrosis and cirrhosis, and HCC in chronic hepatitis B (CHB)^5–7^. A previous study identified that even low-level viremia after achieving virological response was associated with a higher risk of HCC^8^. Considering that treatment modifications in the ETV group were more frequent than those in the TDF group in a previous study^1^, low-level viremia might have acted as a residual confounder which accounts for the different HCC incidence.

Therefore, we investigated the incidence of HCC and death and transplantation in a large number of NA-naïve CHB patients who initiated ETV or TDF, after adjusting multiple confounders including low-level viremia during the treatment course.

## Materials and methods

### Study design and participants

Patients aged 18–80 years, positive for hepatitis B surface antigen (HBsAg) for ≥ 6 months, and not previously treated with NAs, were identified from electronic medical records of CHA Bundang Medical Center, CHA University. Among them, those who started ETV or TDF between 2010 and 2015 were included. Exclusion criteria were treatment < 6 months, seropositivity for other viral infections, previous organ transplantation, occurrence of primary outcomes or seroclearance of HBsAg within 6 months of treatment initiation, and concomitant malignancies diagnosed within 6 months before treatment initiation. Baseline characteristics were collected. Child–Pugh scores and the risk scores for HCC, including Guide with Age, Gender, hepatitis B virus (HBV) DNA, Core promoter mutations and Cirrhosis (GAG-HCC)^9^, Chinese University-HCC (CU-HCC)^10^, and PAGE-B^11^, were calculated.

### Statistical analysis

The follow-up period was calculated as the time from the first prescription of ETV or TDF until the occurrence of the primary outcomes or the last date of follow-up. Follow-up was completed in Dec, 2018. If the treatment regimen was changed, data were censored after 3 months.

The cumulative incidence rate for HCC and death and transplantation was calculated at 1, 3, and 5 years, and throughout the study period. The incidence rate ratio (IRR) were also calculated and compared between the two treatment groups.

Kaplan–Meier estimates were plotted and compared using log-rank test. Cox proportional hazards models were used to calculate the hazard ratio (HR). Subgroup analyses were performed in cirrhotic or elderly patients.

To minimise the effect of potential confounders, we performed propensity score matching and inverse probability of treatment weighting. Details are given in Supplemental Content 1.

## Results

### Patient characteristics

**Figure 1:**
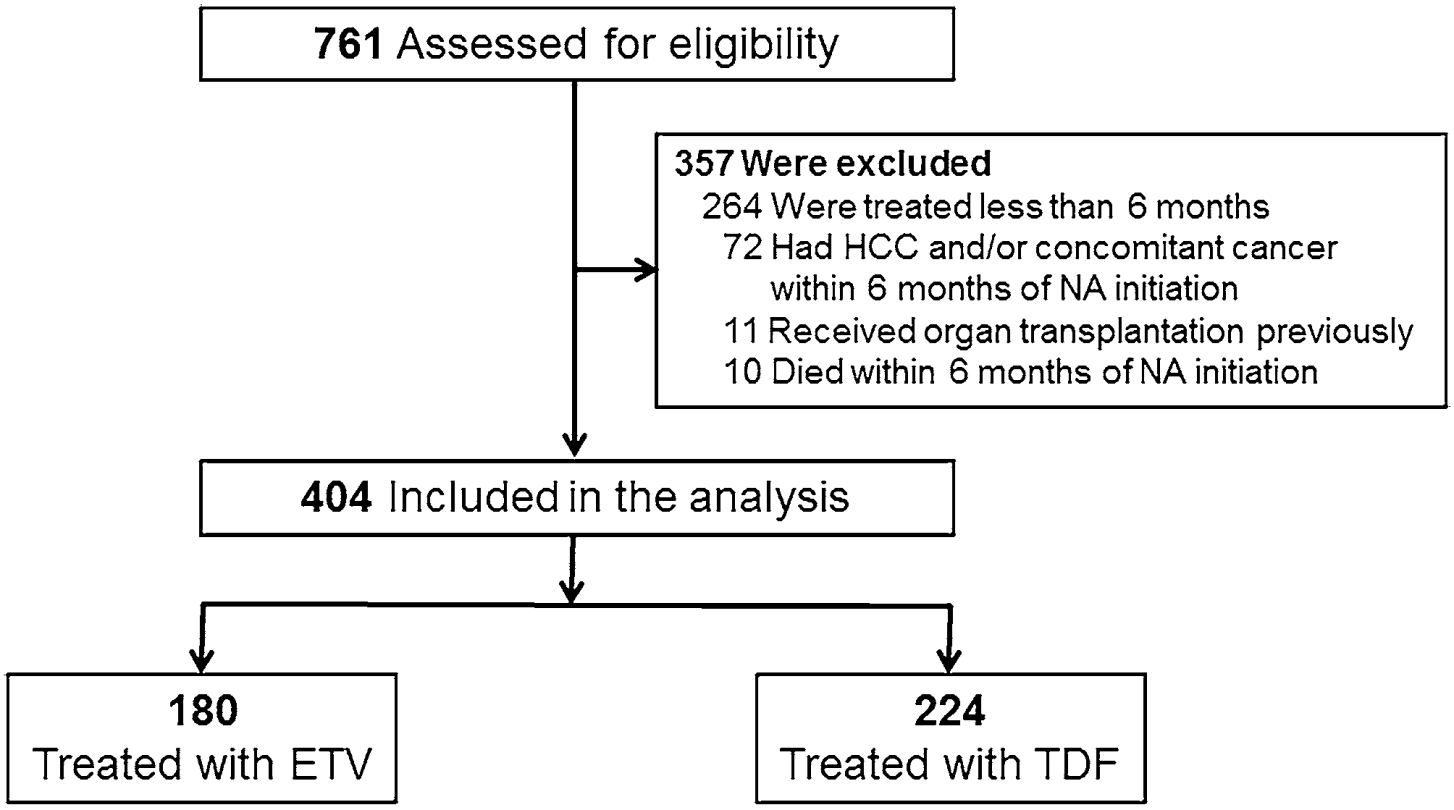
Patient flow.

A total of 761 patients were assessed (Fig. 1) and 404 patients were included in the analysis, after exclusion of 264 patients who were treated for < 6 months, 72 patients who were diagnosed with HCC within 6 months of NA initiation and/or other concomitant cancer(s) within 6 months before NA initiation, 11 patients who underwent organ transplantation, and 10 patients who died within 6 months of NA initiation. In addition, 17 patients had no imaging data since the initiation of NAs; thus excluded for the analysis of HCC incidence. The median follow-up time was 64.0 months [interquartile range (IQR) 30.5–84.3] and 49.1 months (IQR 37.7–62.2) for ETV and TDF, respectively.

The baseline characteristics are summarised in Table 1. A total of 55.9% of the patients were men (58.9% for the ETV vs. 53.6% for the TDF group; *P* = 0.31) and the mean age was 44.9 years (45.4 years for the ETV vs. 44.5 for the TDF group; *P* = 0.51). The median baseline values of laboratory parameters did not show a significant difference.

**Table 1:** Baseline patient characteristics.

Characteristic	ETV (n = 180)	TDF (n = 224)	*P*
Age, mean ± SD, years	45.4 ± 10.8	44.5 ± 11.4	0.51
Male sex, n (%)	106 (58.9)	120 (53.6)	0.31
BMI, kg/m^2^, median (IQR)	23.2 (21.2, 25.6)	23.2 (21.0, 25.5)	0.49
Cirrhosis, n (%)	67 (37.2)	78 (34.8)	0.68
Diabetes, n (%)	23 (12.8)	17 (7.6)	0.10
Hepatitis e antigen positivity, n (%)	118 (67.4)	128 (57.1)	0.038
HBV DNA, log_10_(copies/mL), median (IQR)	7.71 (6.74, 8.64)	7.44 (6.33, 8.53)	0.32
Platelet, × 10^3^/μL, median (IQR)	158 (112, 198)	155 (115, 200)	0.93
Albumin, g/dL, median (IQR)	4.1 (3.7, 4.5)	4.2 (3.8, 4.5)	0.68
Bilirubin, mg/dL, median (IQR)	0.77 (0.54, 1.33)	0.78 (0.55, 1.07)	0.36
PT, INR, median (IQR)	1.06 (1.00, 1.22)	1.06 (1.00, 1.14)	0.30
ALT, IU/L, median (IQR)	83.5 (38.3, 154.0)	85.0 (42.0, 160.5)	0.54
Creatinine, mg/dL, median (IQR)	0.9 (0.8, 1.1)	0.9 (0.7, 1.0)	0.013
Child–Pugh score, median (IQR)	5.0 (5.0, 6.0)	5.0 (5.0, 5.0)	0.012
GAG-HCC score, median (IQR)	85.9 (72.6, 107.8)	82.7 (65.6, 105.9)	0.14
CU-HCC score, median (IQR)	8.5 (7.0, 23.5)	7.0 (4.0, 20.5)	0.31
PAGE-B score, median (IQR)	14.0 (10.0, 16.0)	12.0 (8.0, 16.0)	0.40
**Treatment response at 1 year, n (%)**			
Biochemical	116 (67.4)	132 (62.3)	0.33
Serological^a^	80 (49.7)	93 (49.5)	> 0.99
Virological^b^	117 (68.4)	170 (79.4)	0.018
Sustained virological suppression, n (%)	85 (48.0)	126 (62.4)	0.005
Duration of follow-up, month, median (IQR)	64.0 (30.5, 84.3)	49.1 (37.7, 62.2)	< 0.001

*ETV* entecavir, *TDF* tenofovir disoproxil fumarate, *SD* standard deviation, *IQR* interquartile ranges, *BMI* body mass index, *HBV* hepatitis B virus, *PT* prothrombin time, *INR* international normalized ratio, *ALT* alanine aminotransferase, *GAG-HCC* guide with age, gender, *HBV DNA* core promoter mutations and cirrhosis, *CU* Chinese University.

^a^Hepatitis e antigen seroconversion.

^b^Undetectable HBV DNA by polymerase chain reaction with a detection limit of 10 IU/mL.

Hepatitis e antigen (HBeAg) positivity in the ETV group (67.4%) was significantly higher than that in the TDF group (57.1%; *P* = 0.038). Virological response at 1 year in the ETV group (68.4%) was significantly lower than that in the TDF group (79.4%; *P* = 0.018). Sustained virological suppression was defined as non-detection of HBV DNA after achieving virological response. The proportion of patients with sustained virological suppression in the TDF group (62.4%) was significantly higher than that in the ETV group (48.0%; *P* = 0.005). Biochemical^12^ and serological response at 1 year were comparable.

### Clinical outcomes

#### Entire cohort

HCC developed in 24 patients; 18 and 6 in the ETV and TDF groups, respectively (Table 2). The cumulative incidence rate of HCC at 5 years in the ETV group [2.28 events per 100 person-years; 95% confidence interval (CI) 1.31‒3.63] was significantly higher than that in the TDF group (0.74 events per 100 person-years; 95% CI 0.29‒1.50; *P* = 0.014). Throughout the study period, 2.19 HCCs per 100 person-years (95% CI 1.33‒3.36) in the ETV group occurred vs. 0.71 HCCs per 100 person-years (0.28‒1.44) in the TDF group, with IRR of 3.07 (1.22‒7.74; *P* = 0.012) for ETV over TDF. The risk of HCC was 0.58%, 4.05%, and 8.67% for ETV-treated patients and 0.00%, 2.34%, and 2.80% for TDF-treated patients at 1, 3, and 5 years, respectively.

**Table 2:** Incidence of hepatocellular carcinoma and death and transplantation.

	Hepatocellular carcinoma				Death and transplantation			
	ETV (n = 180)	TDF (n = 224)	IRR (95% CI)	*P*	ETV (n = 180)	TDF (n = 224)	IRR (95% CI)	*P*
**1 year**								
Cumulative incidence	1	0			0	0		
CIR (95% CI), per 100 PY	0.58 (0.03‒2.55)	0.00 (0.00‒0.00)	‒	0.27	0.00 (0.00‒0.00)	0.00 (0.00‒0.00)	‒	‒
**3 years**								
Cumulative incidence	7	5			1	2		
CIR (95% CI), per 100 PY	1.53 (0.66‒2.95)	0.85 (0.30‒1.82)	1.81 (0.57‒5.69)	0.31	0.21 (0.01‒0.90)	0.32 (0.05‒0.97)	0.65 (0.06‒7.20)	0.73
**5 years**								
Cumulative incidence	15	6			2	2		
CIR (95% CI), per 100 PY	2.28 (1.31‒3.63)	0.74 (0.29‒1.50)	3.08 (1.19‒7.93)	0.014	0.28 (0.05‒0.86)	0.23 (0.04‒0.70)	1.23 (0.17‒8.74)	0.83
**Total period**								
Cumulative incidence	18	6			4	2		
CIR (95% CI), per 100 PY	2.19 (1.33‒3.36)	0.71 (0.28‒1.44)	3.07 (1.22‒7.74)	0.012	0.45 (0.14‒1.05)	0.23 (0.04‒0.70)	1.99 (0.37‒10.88)	0.42

*ETV* entecavir, *TDF* tenofovir disoproxil fumarate, *CIR* cumulative incidence rate, *IRR* incidence rate ratio, *CI* confidence interval, *PY* person-years.

In contrast, death and transplantation was not significantly different (Table 2). The cumulative incidence rate of death and transplantation per 100 person-years (95% CI) was 0.45 (0.14‒1.05) and 0.23 (0.04‒0.70) for the ETV and TDF groups, respectively, with the IRR of 1.99 (0.37‒10.88; *P* = 0.42) for ETV over TDF. The risk of death and transplantation was 0.00%, 0.56%, and 1.11% for the ETV group and 0.00%, 0.89%, and 0.89% for the TDF group at 1, 3, and 5 years, respectively.

The univariate analysis revealed that TDF was associated with the lower incidence of HCC (HR, 0.31; 95% CI, 0.12‒0.79; *P* = 0.014; Supplemental Contents 2a and 3). However, for death and transplantation, TDF was not a predictive factor (HR, 0.53; 95% CI, 0.09‒2.98; *P* = 0.47; Supplemental Content 2b and 3).

Because of the low number of events, we proceeded to propensity score matching and inverse probability of treatment weighting rather than performing multivariate analyses.

#### Propensity score-matched cohort

Propensity score matching yielded 168 and 175 pairs of patients for the evaluation of HCC and death and transplantation, respectively (Supplemental Content 4). The risk of HCC in the TDF group was lower than that in the ETV group (Supplemental Content 5a; HR 0.27; 95% CI 0.08‒0.98; *P* = 0.046). However, the percentage of patients with sustained virological suppression remained significantly different after matching (44.8% in the ETV group vs. 57.8% in the TDF group; standardised mean difference [SMD], 0.26; Supplemental Content 4).

Death and transplantation did not differ (Supplemental Content 5b; HR, 1.00, 95% CI 0.06‒15.99; *P* > 0.99).

#### Inverse probability of treatment-weighted cohort

Supplemental Content 6 summarises the baseline characteristics of the patients after inverse probability of treatment weighting.

The risk of HCC in the TDF group was lower than that in the ETV group (HR 0.32; 95% CI 0.13‒0.80; *P* = 0.015), whereas patients treated with ETV or TDF had comparable outcomes in terms of death and transplantation (HR 0.49, 95% CI 0.08‒3.14; *P* = 0.45; Supplemental Content 7).

However, patients with sustained virological suppression (46.6% in the ETV group vs. 58.4% in the TDF group;   SMD, 0.24) remained significantly imbalanced after weighting (Supplemental Content 6).

### Effect of sustained virological suppression on clinical outcomes

**Figure 2:**
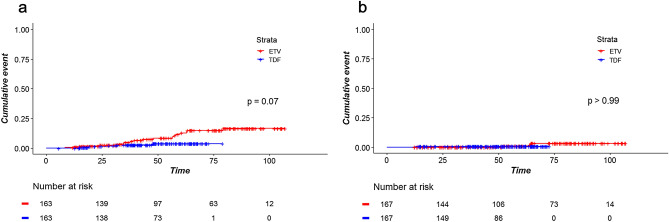
Time-to-event curves for (**a**) hepatocellular carcinoma and (**b**) death and transplantation in a cohort of patients matched for propensity scores including sustained virological suppression as a matching variable.

Because a significant difference was observed in ‘sustained virological suppression’ between the two groups after matching and weighting, we assumed that sustained virological suppression can be a potential confounder that might have influenced the result. Therefore, sustained virological suppression was additionally included in the matching variables for propensity score matching. After matching, the proportion of patients with sustained virological suppression did not differ (44.8% in the ETV group vs. 52.8% in the TDF group; *P* = 0.18). In this matched cohort, the TDF group showed lower incidence of HCC (HR 0.36; 95% CI 0.12‒1.14; Fig. 2a); however, the statistical significance was not reached (*P* = 0.08). Both drugs showed a comparable risk of death and transplantation (Fig. 2b; HR 1.00; 95% CI 0.06‒15.99; *P* > 0.99).

**Figure 3:**
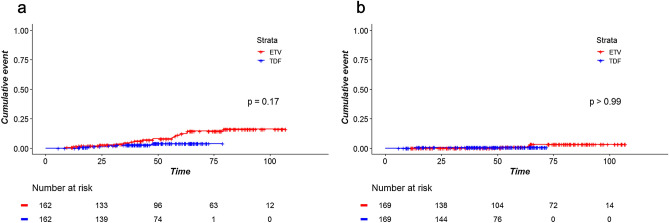
Time-to-event curves for (**a**) hepatocellular carcinoma and (**b**) death and transplantation in a subcohort of patients with no treatment modification matched for propensity scores including sustained virological suppression as a matching variable.

Subsequently, the analyses excluding patients whose treatment regimen was changed during the study period showed that TDF was not significantly associated with lower incidence of HCC and death and transplantation in the matched cohort (Fig. 3).

Inverse probability of treatment weighting, additionally including the presence of sustained virological suppression, did not produce a balanced cohort (sustained virological suppression, 46.9% in the ETV group vs. 56.1% in the TDF group; *P* = 0.038); therefore reanalyses were not performed.

### Cirrhotic subgroup

The baseline characteristics of cirrhotic patients (145 [35.9%]) before and after matching are shown in Supplemental Content 8.

HCC occurred in 16 and 5 patients in the ETV and the TDF groups, respectively. The HR of TDF was 0.30 (95% CI, 0.11‒0.84; *P* = 0.021) in the univariate analysis (Supplemental Content 9). Death and transplantation outcome did not differ (HR of TDF, 0.58; 95% CI, 0.10‒3.25; *P* = 0.53).

**Figure 4:**
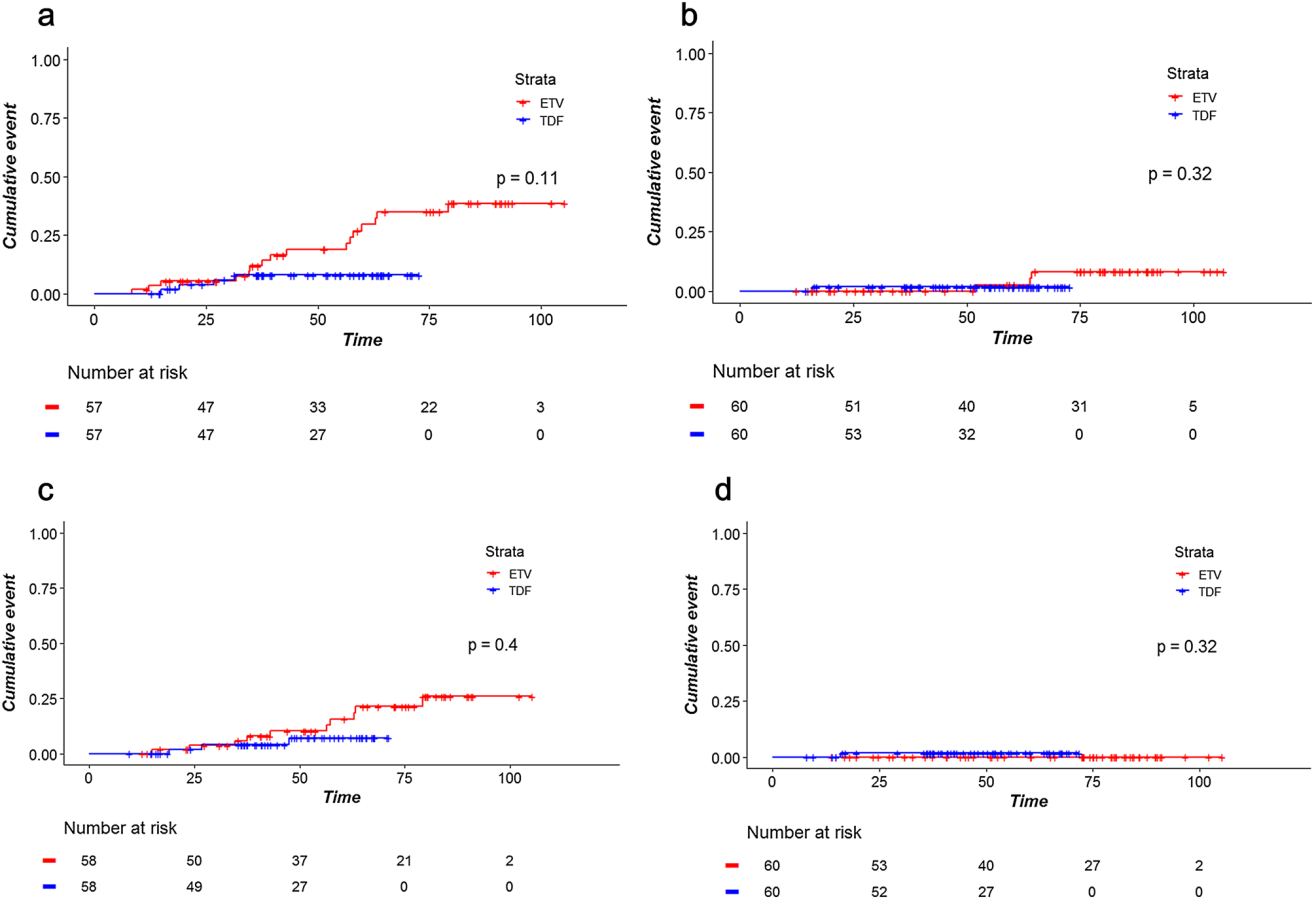
Time-to-event curves for (**a**) hepatocellular carcinoma and (**b**) death and transplantation in a subcohort of patients with cirrhosis matched for propensity scores including sustained virological suppression as a matching variable; and (**c**) hepatocellular carcinoma and (**d**) death and transplantation in a subcohort of patients ≥ 50 years matched for propensity scores including sustained virological suppression as a matching variable.

Figure 4 shows the incidence of primary outcomes according to the treatment regimen in the matched cohort. TDF-treated patients showed lower risk of HCC with marginal statistical significance (HR 0.36; 95% CI 0.12‒1.14; *P* = 0.08; Fig. 4a). The death and transplantation outcomes were comparable (HR, 1.00; 95% CI, 0.06‒15.99; *P* > 0.99; Fig. 4b).

### Elderly subgroup

A total of 52.2% of the ETV group (94 of 180 patients) and 29.5% of TDF group (66 of 224 patients) were treated ≥ 5 years. Among them, 3 patients developed HCC, and all were treated with ETV and were older than 50 years when ETV was initiated (Supplemental Content 10).

The incidence of HCC was not significantly associated with TDF treatment on the univariate analysis (HR 0.41; 95% CI 0.13‒1.35; *P* = 0.14; Supplemental Content 11). Death and transplantation was comparable (HR, 1.54; 95% CI, 0.14‒17.01; *P* = 0.72). After matching, the risk of HCC was marginally lower in the TDF group (HR 0.36; 95% CI 0.12‒1.14; *P* = 0.08; Fig. 4c). Death and transplantation (HR 1.00; 95% CI 0.06‒15.99; *P* > 0.99; Fig. 4d) did not differ between the two groups.

## Discussion

This study compared the impact of two NAs currently recommended as first-line therapy for CHB^5,12,13^, ETV and TDF, on the incidence of HCC and death and transplantation. TDF was associated with lower incidence of HCC; however, the impact (HRs) and statistical significance (*P* values) were blunted when sustained virological suppression was additionally controlled as a potential confounder. These findings were reproduced in the subgroup analyses including patients with cirrhosis and those older than 50 years.

We analysed a large cohort of CHB patients newly treated with ETV or TDF by using robust statistical methods. Particularly, we assessed sustained virological suppression in every individual. Previous literature reported that not only the virological response at a specific time point^6,7^ but also low-level viremia during NA treatment^8^ was associated with higher risk of HCC. However, previous studies investigating the impact of TDF on the incidence of HCC did not assess dynamic change of viral replication status during the treatment period^1,2^. In addition, we selected patients prescribed with the either drug from 2010 to minimise the effect of unmeasured potential confounders, such as difference in medical practice that may vary by time. Finally, the long duration of follow-up allowed us to provide comprehensive data regarding the clinical outcomes.

The use of potent NAs reduced hepatic inflammation, progression of fibrosis, and development of cirrhosis and decompensation that could accompany with viral replication^5–7,12,13^. Due to the improvement in acute/subacute liver-related complications and life expectancy, HCC is now a major health-related problem in CHB patients^14^. Accordingly, recent studies have focused on whether one drug has an advantage over another in long-term outcomes, such as HCC and death and transplantation^1–4^.

Since Choi et al*.* reported that TDF, in comparison with ETV, reduced HCC by analysing large insurance claim data and hospital cohorts^1^, subsequent studies have analysed the association between NAs and the incidence of HCC. However, they have failed to show consistent results^15–22^. Of note, a recent meta-analysis including more than 60,000 patients from 15 studies demonstrated that TDF significantly reduced the risk of HCC by 20%^23^. Similarly, the ETV group showed a higher incidence of HCC compared to the TDF group in our crude analysis, approximately from 3 years after initiation of therapy. Two kinds of parameters were considered to adjust virological confounders: (1) virological response at 1 year and (2) sustained virological suppression (continuously undetectable HBV DNA after virological response at 1 year). When we included ‘virological response at 1 year’ in the matching model similar to the previous study^1^, TDF group showed significantly lower incidence of HCC (data not shown). However, the difference became marginally significant when ‘sustained virological suppression’ was vicariously used and adjusted by matching. Similarly, after excluding patients whose treatment regimen was changed, the TDF group showed a lower incidence of HCC however without statistical significance. Consistently, TDF was associated with lower HCC in patients with cirrhosis; however, the statistical significance was not reached. Overall, these results indicate that complete and continuous virological suppression is important in the development of HCC.

Previous study including Caucasian patients receiving ETV or TDF demonstrated that all patients who developed HCC beyond 5 years of treatment were older than 50 years when NAs were initiated^24^. In our study, only 3 patients developed HCC beyond 5 years; all were administered ETV and were older than 50 years at the first prescription date. Although ETV group showed higher incidence of HCC, further observation is warranted considering the small number of events and lower number of patients treated more than 5 years in the TDF group (29.5% vs. 52.2% in the ETV group).

One caveat is that the incidence curve for HCC shows significant separation approximately from 3 years of treatment. Indeed, although the statistical significance was not reached after adjustment of the ‘sustained virological suppression’, the impact of TDF on the incidence of HCC was still considerable (64% reduction in risk of HCC, as compared to ETV). Regarding the substantial range of 95% CI in our analysis, a validation study with a larger number of patients is needed in the future. Meanwhile, HCC increases at approximately 4 years in the ETV group, before reaching a plateau after 6 years. In contrast, TDF group did not show a remarkable increase in HCC until up to 6 years. Considering relatively shorter time of treatment for TDF, it is crucial to follow-up these patients for a longer period to check late HCC occurrence.

The major limitation of this study is that we evaluated retrospective data; therefore, the treatment was not based on randomised assignment. Although we tried to minimise the effect of confounding variables by utilising robust statistical methodologies, we cannot completely exclude the impact of unmeasured confounders, such as those influenced the selection of a drug for each patient. Second, although we included more than 400 patients and used robust statistical methodology, the sample size might not have been large enough to lead to a firm conclusion on the choice between ETV and TDF. Further validation studies with larger cohort are definitely needed to examine and determine the impact of TDF on the incidence of HCC, after controlling the virological factor, such as sustained virological suppression. Third, adherence to the prescription was assessed from the review of the medical records. Although > 75% of patients had appropriate records for evaluation of the adherence, the possibility of missing or inaccurate data still exists. However, assessment of sustained virological suppression in every patient might have resolved the issue, at least in part. Given the limited number of studies and inconsistent results so far^1–4,25–32^, a report of outcomes from extended follow-up as well as from various ethnic groups is definitely warranted.

## Conclusion

We showed that TDF was significantly associated with lower incidence of HCC compared to ETV. However, the difference became less significant after adjustment for the presence of sustained virological suppression, suggesting the importance of maintaining complete and continuous virological suppression in the prevention of HCC. Future larger studies comparing the two drugs in the context of sustained virological suppression over a longer period of time are warranted.

### Ethics approval and consent to participate

All procedures were in accordance with the ethical standards of the responsible committee on human experimentation (institutional and national) and with the Helsinki Declaration of 1975, as revised in 2008. The written informed consent was waived due to the retrospective design of the study. The institutional review board of the CHA Bundang Medical Center granted a waiver of informed consent and approval of this observational study with deidentified data (approval No. 2019-07-032).

## Supplementary information

Supplementary file 1.

## Data Availability

The datasets used and/or analysed during the current study are available from the corresponding author on reasonable request.

## Abbreviations

TDF: Tenofovir disoproxil fumarate
ETV: Entecavir
HCC: Hepatocellular carcinoma
NA: Nucleos(t)ide analogue
CHB: Chronic hepatitis B
HBsAg: Hepatitis B surface antigen
HBV: Hepatitis B virus
GAG-HCC: Guide with Age, Gender, HBV DNA, Core promoter mutations and Cirrhosis
CU: Chinese University
IRR: Incidence rate ratio
HR: Hazard ratio
IQR: Interquartile ranges
HBeAg: Hepatitis e antigen
CI: Confidence interval

## References

[1] Choi J (2019). Risk of hepatocellular carcinoma in patients treated with entecavir vs tenofovir for chronic hepatitis B: a Korean nationwide cohort study. JAMA Oncol..

[2] Kim SU (2019). A multi-center study of entecavir vs. tenofovir on prognosis of treatment-naive chronic hepatitis B in the Republic of Korea. J. Hepatol..

[3] Wang X (2019). Nucleos(t)ide analogues for reducing hepatocellular carcinoma in chronic hepatitis B patients: a systematic review and meta-analysis. Gut Liver.

[4] Zhang Z, Zhou Y, Yang J, Hu K, Huang Y (2019). The effectiveness of TDF versus ETV on incidence of HCC in CHB patients: a meta analysis. BMC Cancer.

[5] EASL (2017). Clinical Practice Guidelines on the management of hepatitis B virus infection. J. Hepatol..

[6] Zoutendijk R (2013). Virological response to entecavir is associated with a better clinical outcome in chronic hepatitis B patients with cirrhosis. Gut.

[7] Papatheodoridis GV, Chan HL, Hansen BE, Janssen HL, Lampertico P (2015). Risk of hepatocellular carcinoma in chronic hepatitis B: assessment and modification with current antiviral therapy. J. Hepatol..

[8] Kim JH (2017). Low-level viremia and the increased risk of hepatocellular carcinoma in patients receiving entecavir treatment. Hepatology.

[9] Yuen MF (2009). Independent risk factors and predictive score for the development of hepatocellular carcinoma in chronic hepatitis B. J. Hepatol..

[10] Wong VW (2010). Clinical scoring system to predict hepatocellular carcinoma in chronic hepatitis B carriers. J. Clin. Oncol..

[11] Papatheodoridis G (2016). PAGE-B predicts the risk of developing hepatocellular carcinoma in Caucasians with chronic hepatitis B on 5-year antiviral therapy. J. Hepatol..

[12] Terrault NA (2018). Update on prevention, diagnosis, and treatment of chronic hepatitis B: AASLD 2018 hepatitis B guidance. Hepatology.

[13] KASL clinical practice guidelines for management of chronic hepatitis B. *Clin. Mol. Hepatol.* **25**, 93–159, 10.3350/cmh.2019.1002 (2019). 10.3350/cmh.2019.1002 PMC6589848 31185710

[14] Choi J, Han S, Kim N, Lim YS (2017). Increasing burden of liver cancer despite extensive use of antiviral agents in a hepatitis B virus-endemic population. Hepatology.

[15] Dave S (2020). Comparative effectiveness of entecavir vs tenofovir for preventing hepatocellular carcinoma in patients with chronic hepatitis B: a systematic review and meta-analysis. Hepatology.

[16] Liu K (2019). Tenofovir disoproxil fumarate reduces hepatocellular carcinoma, decompensation and death in chronic hepatitis B patients with cirrhosis. Aliment. Pharmacol. Ther..

[17] Yip TC (2020). Tenofovir is associated with lower risk of hepatocellular carcinoma than entecavir in patients with chronic HBV infection in China. Gastroenterology.

[18] Chen MB (2019). Comparative efficacy of tenofovir and entecavir in nucleos(t)ide analogue-naive chronic hepatitis B: a systematic review and meta-analysis. PLoS ONE.

[19] Li M (2020). Tenofovir versus entecavir in lowering the risk of hepatocellular carcinoma development in patients with chronic hepatitis B: a critical systematic review and meta-analysis. Hepatol. Int..

[20] Hsu YC (2020). Tenofovir versus entecavir for hepatocellular carcinoma prevention in an international consortium of chronic hepatitis B. Am. J. Gastroenterol..

[21] Lee SW (2020). Comparison of tenofovir and entecavir on the risk of hepatocellular carcinoma and mortality in treatment-naïve patients with chronic hepatitis B in Korea: a large-scale, propensity score analysis. Gut.

[22] Oh H (2020). No difference in incidence of hepatocellular carcinoma in patients with chronic hepatitis B virus infection treated with entecavir vs tenofovir. Clin. Gastroenterol. Hepatol..

[23] Choi WM, Choi J, Lim YS (2020). Effects of tenofovir vs entecavir on risk of hepatocellular carcinoma in patients with chronic HBV infection: a systematic review and meta-analysis. Clin. Gastroenterol. Hepatol..

[24] Papatheodoridis GV (2017). The risk of hepatocellular carcinoma is decreasing after the first 5 years of entecavir or tenofovir in Caucasians with chronic hepatitis B. Hepatology.

[25] Kim BG (2018). Mortality, liver transplantation and hepatic complications in patients with treatment-naive chronic hepatitis B treated with entecavir vs tenofovir. J. Viral Hepat..

[26] Kim YM (2018). Real-world single-center experience with entecavir and tenofovir disoproxil fumarate in treatment-naïve and experienced patients with chronic hepatitis B. Saudi J. Gastroenterol..

[27] Tsai MC (2017). Long-term outcomes of hepatitis B virus-related cirrhosis treated with nucleos(t)ide analogs. J. Formos. Med. Assoc..

[28] Yu JH, Jin YJ, Lee JW, Lee DH (2018). Remaining hepatocellular carcinoma risk in chronic hepatitis B patients receiving entecavir/tenofovir in South Korea. Hepatol. Res..

[29] Coffin CS (2014). The incidence of hepatocellular carcinoma is reduced in patients with chronic hepatitis B on long-term nucleos(t)ide analogue therapy. Aliment. Pharmacol. Ther..

[30] Goyal SK (2015). Prolonged use of tenofovir and entecavir in hepatitis B virus-related cirrhosis. Indian J. Gastroenterol..

[31] Idilman R (2015). Long-term entecavir or tenofovir disoproxil fumarate therapy in treatment-naive chronic hepatitis B patients in the real-world setting. J. Viral Hepat..

[32] Riveiro-Barciela M (2017). Effectiveness and safety of entecavir or tenofovir in a Spanish cohort of chronic hepatitis B patients: validation of the page-B score to predict hepatocellular carcinoma. Dig. Dis. Sci..

