# Supplementary material for: Hepatocellular carcinoma and death and transplantation in chronic hepatitis B treated with entecavir or tenofovir disoproxil fumarate
Source: Sci Rep. 2020 Aug 11;10:13537. doi: 10.1038/s41598-020-70433-z (PMC7419516; doi:10.1038/s41598-020-70433-z)
Supplement: Supplementary file 1 [file 41598_2020_70433_MOESM1_ESM.docx]

**Title:** Hepatocellular carcinoma and death and transplantation in chronic hepatitis B treated with entecavir or tenofovir disoproxil fumarate

**Running Title:** Liver cancer and nucleos(t)ide analogues

**Authors:** Yeonjung Ha (YH), MD, PhD^a,*^, Young Eun Chon (YEC), MD, PhD^a^, Mi Na Kim (MNK), MD, PhD^a^, Joo Ho Lee (JHL), MD, PhD^a^, Seong Gyu Hwang (SGH), MD, PhD^a^

**Affiliations:** ^a^Department of Gastroenterology, CHA Bundang Medical Center, CHA University, 59 Yatap-ro, Bundang-gu, Seongnam-si, Gyeonggi-do 13496, South Korea

***Corresponding author:**

Yeonjung Ha, MD, PhD

Department of Gastroenterology, CHA Bundang Medical Center, CHA University, 59 Yatap-ro, Bundang-gu, Seongnam-si, Gyeonggi-do 13496, South Korea; Tel: +82-31-780-2947; Fax: +82-31-780-5221; E-mail: [yeonjung.ha@gmail.com](mailto:yeonjung.ha@gmail.com)

**Supplemental Content 1**

A one-to-one optimal matching with propensity score from the logistic regression was conducted. The variables for matching that were selected based on their *a priori* possibility of confounding were as follows: age, sex, presence of cirrhosis, presence of diabetes, hepatitis e antigen positivity, hepatitis B virus (HBV) DNA, platelet, albumin, total bilirubin, prothrombin time, alanine aminotransferase, creatinine, Child–Pugh score, GAG-HCC score, CU-HCC score, and PAGE-B score. Sustained virological suppression, defined as non-detection of HBV DNA after achieving virological response, was additionally adjusted in the subsequent analyses.

In the inverse probability of treatment weighting, the propensity scores calculated above were used to weigh each patient, with the aim of balancing the baseline variables between the two groups. Post-weighting balance checked.

The Kaplan–Meier curves were compared by stratified and weighted log-rank test in the analyses using propensity score-matched and inverse probability of treatment-weighted cohort, respectively. Similarly, stratified and weighted Cox proportional hazards models were utilized to estimate the HRs of the treatment regimen on the occurrence of primary outcomes in the matched and weighted cohort.

Missing data on baseline laboratory variables were imputed using predictive mean matching via multiple imputation by chained equations under the missing at random assumption.^1^ The variables eligible for imputation were platelet, albumin, total bilirubin, PT, and creatinine, which comprised 2·5%, 1·7%, 1·2%, 2·2%, and 1·7% of the entire data, respectively. Unless noted, multiple imputed data sets were used for analyses.

All statistical analyses were performed using RStudio 1·1·463 ([www.rstudio.com](http://www.rstudio.com))^2^ with the IPWsurvival,^3^ MatchIt,^4^ MICE,^1^ survival,^5^ and survminer packages.^6^ All *P*-values are two-tailed, and *P* <0·05 was considered statistically significant.

**Reference**

1. Buuren Sv, Groothuis-Oudshoorn K. mice: Multivariate imputation by chained equations in R. Journal of statistical software 2010:1-68.

2. RStudio Team (2016). RStudio: Integrated Development for R. RStudio I, Boston, MA URL, http://www.rstudio.com/.

3. Le Borgne F, Foucher Y, Foucher MY. Package ‘IPWsurvival’. 2014.

4. King G, Ho D, Stuart EA, et al. MatchIt: nonparametric preprocessing for parametric causal inference. 2011.

5. Therneau TM, Grambsch PM. Modeling survival data: extending the Cox model: Springer Science & Business Media, 2013.

6. Kassambara A, Kosinski M, Biecek P. survminer: Drawing Survival Curves using'ggplot2'. R package version 0.3 2017;1.

**Supplemental Content 2 (a)**


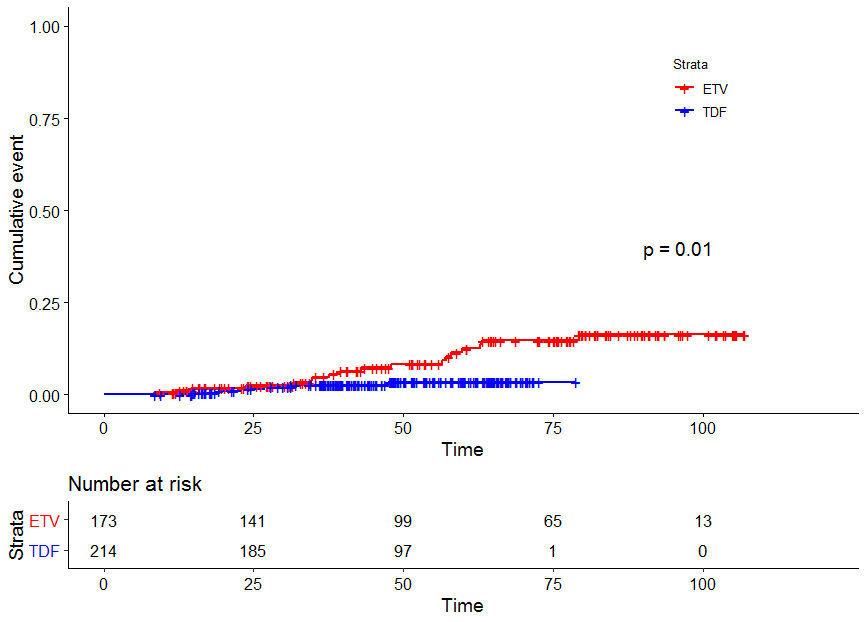


**Supplemental Content 2 (b)**


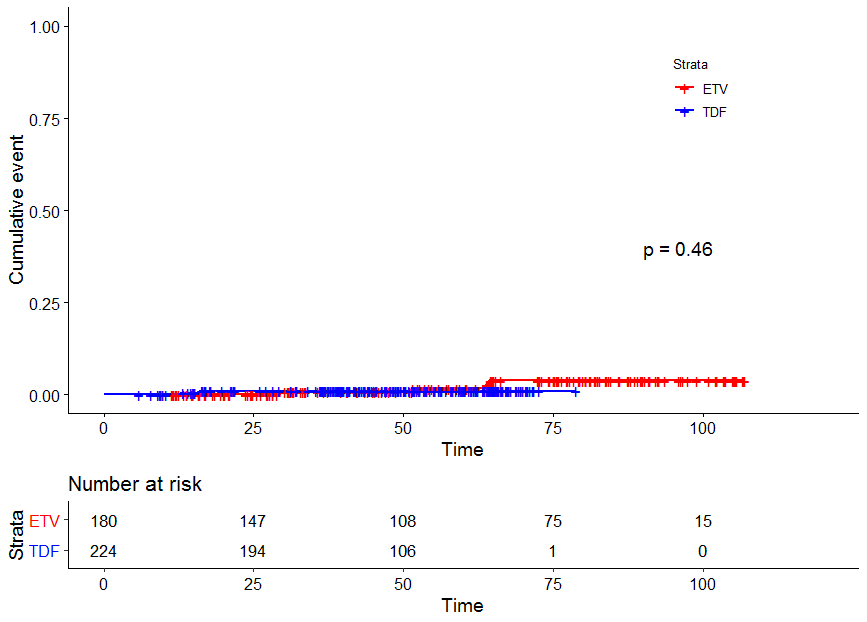


**Supplemental Content 3**. Risk of hepatocellular carcinoma or death in entire cohort on univariate analysis

| Variable | Risk of HCC | | Risk of  death and transplantation | |
| --- | --- | --- | --- | --- |
|  | Hazard ratio (95% CI) | *P* | Hazard ratio (95% CI) | *P* |
| TDF treatment | 0.31 (0.12‒0.79) | 0.014 | 0.53 (0.09‒2.98) | 0.47 |
| Age, per 1 year | 1.07 (1.03‒1.11) | <0.001 | 1.02 (0.95‒1.10) | 0.52 |
| Male sex | 1.41 (0.62‒3.22) | 0.42 | 56.36 (0.08‒Inf) | 0.23 |
| BMI, per 1 kg/m^2^ | 1.12 (0.95‒1.31) | 0.18 | 1.30 (0.98‒1.71) | 0.07 |
| Cirrhosis | 13.15 (3.92‒44.08) | <0.001 | 127.80 (0.11‒Inf) | 0.18 |
| Diabetes | 8.64 (3.83‒19.51) | <0.001 | 4.53 (0.83‒24.75) | 0.08 |
| Hepatitis e antigen | 0.62 (0.28‒1.39) | 0.25 | 0.95 (0.16‒5.68) | 0.95 |
| HBV DNA, per 1 log_10_(copies/mL) | 0.98 (0.71‒1.36) | 0.92 | 0.92 (0.49‒1.74) | 0.81 |
| Platelet, per 1,000/μL | 0.99 (0.98‒1.00) | 0.002 | 0.98 (0.96‒0.99) | 0.011 |
| Albumin, per 1 g/dL | 0.38 (0.22‒0.66) | <0.001 | 0.14 (0.05‒0.37) | <0.001 |
| Bilirubin, per 1 mg/dL | 1.00 (0.92‒1.10) | 0.98 | 1.02 (0.91‒1.13) | 0.75 |
| PT, per 1 INR | 2.93 (0.98‒8.82) | 0.06 | 5.86 (1.42‒24.15) | 0.014 |
| ALT, per 1 IU/L | 1.00 (0.99‒1.00) | 0.09 | 0.97 (0.93‒1.00) | 0.046 |
| Creatinine, per 1 mg/dL | 1.10 (0.32‒3.77) | 0.88 | 2.28 (0.81‒6.41) | 0.12 |
| Child–Pugh score, per 1 point | 1.34 (1.08‒1.66) | 0.007 | 1.88 (1.42‒2.51) | <0.001 |
| Biochemical response | 0.69 (0.31‒1.56) | 0.37 | 0.76 (0.13‒4.55) | 0.76 |
| Serological response* | 0.55 (0.23‒1.34) | 0.19 | 0.59 (0.10‒3.55) | 0.57 |
| Virological response† | 0.75 (0.31‒1.81) | 0.52 | 0.63 (0.12‒3.43) | 0.59 |
| Sustained virological suppression§ | 1.46 (0.65‒3.30) | 0.36 | 0.29 (0.03‒2.56) | 0.26 |

* Hepatitis e antigen seroconversion.

† Undetectable HBV DNA by polymerase chain reaction with a detection limit of 10 IU/mL.

HCC, hepatocellular carcinoma; CI, confidence interval; TDF, tenofovir disoproxil fumarate; BMI, body mass index; HBV, hepatitis B virus; PT, prothrombin time; INR, international normalized ratio; ALT, alanine aminotransferase.

§ Extended Cox proportional hazards model for time-dependent covariate.

**Supplemental Content 4**. Baseline patient characteristics after propensity score matching

|  | Cohort for hepatocellular carcinoma | | | Cohort for death and transplantation | | |
| --- | --- | --- | --- | --- | --- | --- |
| Characteristic | ETV  (n=168) | TDF  (n=168) | SMD | ETV  (n=175) | TDF  (n=175) | SMD |
| Age, mean ± SD, years | 45.4 ± 10.9 | 45.0 ± 11.6 | 0.03 | 45.4 ± 11.0 | 44.0 ± 11.7 | 0.12 |
| Male sex, n (%) | 100 (59.5) | 94 (56.0) | 0.07 | 103 (58.9) | 99 (56.6) | 0.05 |
| BMI, kg/m^2^, median (IQR) | 23.2  (21.3, 25.6) | 23.2  (21.1, 25.5) | 0.02 | 23.2  (21.2, 25.6) | 23.4  (21.2, 25.8) | 0.04 |
| Cirrhosis, n (%) | 58 (34.5) | 56 (33.3) | 0.03 | 63 (36.0) | 54 (30.9) | 0.11 |
| Diabetes, n (%) | 21 (12.5) | 15 (8.9) | 0.12 | 22 (12.6) | 16 (9.1) | 0.11 |
| Hepatitis e antigen positivity, n (%) | 111 (66.1) | 109 (64.9) | 0.03 | 118 (67.4) | 117 (66.9) | 0.01 |
| HBV DNA, log_10_(copies/mL), median (IQR) | 7.82  (6.85, 8.64) | 7.74  (6.48, 8.74) | 0.04 | 7.80  (6.79, 8.65) | 7.70  (6.57, 8.77) | 0.01 |
| Platelet, ×10^3^/μL, median (IQR) | 159  (113, 198) | 153  (112, 198) | 0.03 | 159  (113, 199) | 158  (115, 206) | 0.02 |
| Albumin, g/dL, median (IQR) | 4.2 (3.7, 4.5) | 4.2 (3.8, 4.4) | 0.03 | 4.1 (3.7, 4.5) | 4.2 (3.8, 4.5) | 0.04 |
| Bilirubin, mg/dL, median (IQR) | 0.79  (0.54, 1.32) | 0.80  (0.58, 1.16) | 0.11 | 0.77  (0.54, 1.30) | 0.79  (0.56, 1.14) | 0.11 |
| PT, INR, median (IQR) | 1.06  (1.00, 1.21) | 1.07  (1.00, 1.15) | 0.12 | 1.06  (1.00, 1.21) | 1.06  (1.00, 1.14) | 0.12 |
| ALT, IU/L, median (IQR) | 86.0  (38.8, 155.3) | 84.0  (42.8, 156.3) | 0.02 | 86.0  (39.5, 154.5) | 85.0  (42.5, 156.5) | 0.02 |
| Creatinine, mg/dL, median (IQR) | 0.9 (0.8, 1.1) | 0.9 (0.8, 1.0) | 0.11 | 0.9 (0.8, 1.1) | 0.9 (0.8, 1.0) | 0.09 |
| Child–Pugh score, median (IQR) | 5.0 (5.0, 6.0) | 5.0 (5.0, 5.0) | 0.11 | 5.0 (5.0, 6.0) | 5.0 (5.0, 5.0) | 0.11 |
| GAG-HCC score, median (IQR) | 85.5  (72.0, 105.1) | 84.0  (68.9, 105.6) | 0.07 | 85.9  (72.6, 105.4) | 82.4  (66.5, 104.2) | 0.15 |
| CU-HCC score, median (IQR) | 5.5  (4.0, 23.5) | 7.0  (4.0, 20.9) | 0.05 | 5.5  (4.0, 22.0) | 5.5  (4.0, 19.8) | 0.08 |
| PAGE-B score, median (IQR) | 13.0  (10.0, 16.0) | 12.0  (10.0, 17.0) | 0.05 | 13.0  (10.0, 16.0) | 12.0  (8.0, 16.0) | 0.11 |
| Treatment response at 1 year, n (%) |  |  |  |  |  |  |
| Biochemical | 109 (67.3) | 97 (59.2) | 0.17 | 113 (67.7) | 104 (63.0) | 0.10 |
| Serological* | 78 (50.7) | 82 (56.6) | 0.12 | 80 (51.0) | 89 (61.4) | 0.21 |
| Virological† | 111 (68.1) | 128 (77.1) | 0.20 | 113 (68.1) | 128 (76.7) | 0.19 |
| Sustained virological suppression, n (%) | 73 (44.8) | 96 (57.8) | 0.26 | 69 (41.6) | 89 (53.6) | 0.24 |
| Duration of follow-up, month, median (IQR) | 64.8  (33.1, 84.5) | 49.6  (38.5, 62.1) | 0.54 | 64.0  (31.0, 84.2) | 49.7  (38.1, 62.2) | 0.49 |

* Hepatitis e antigen seroconversion.

† Undetectable HBV DNA by polymerase chain reaction with a detection limit of 10 IU/mL.

ETV, entecavir; TDF, tenofovir disoproxil fumarate; SMD, standardized mean difference; SD, standard deviation; IQR, interquartile ranges; BMI, body mass index; HBV, hepatitis B virus; PT, prothrombin time; INR, international normalized ratio; ALT, alanine aminotransferase; GAG, guide with age, gender, HBV DNA, core promoter mutations and cirrhosis; CU, Chinese University.

**Supplemental Content 5 (a)**

**
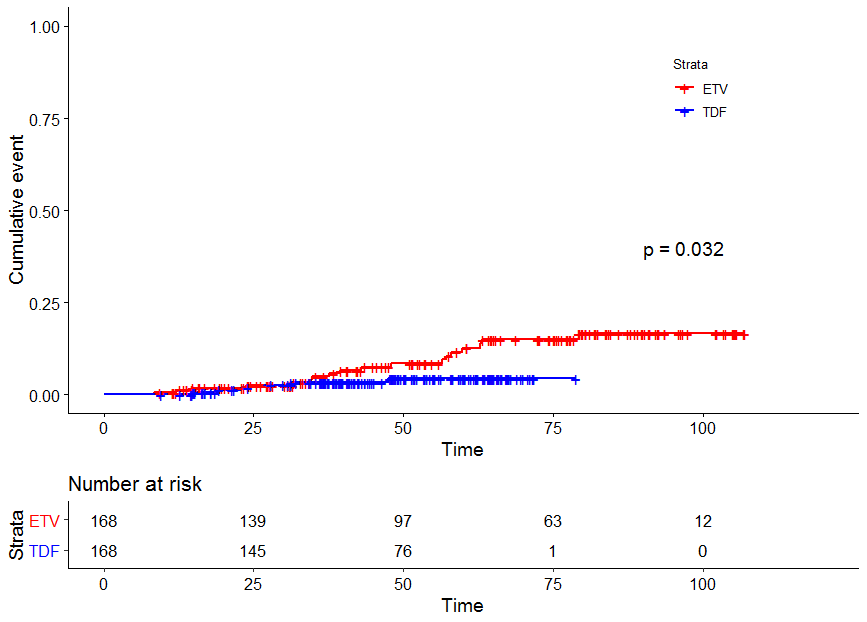
**

**Supplemental Content 5 (b)**

**
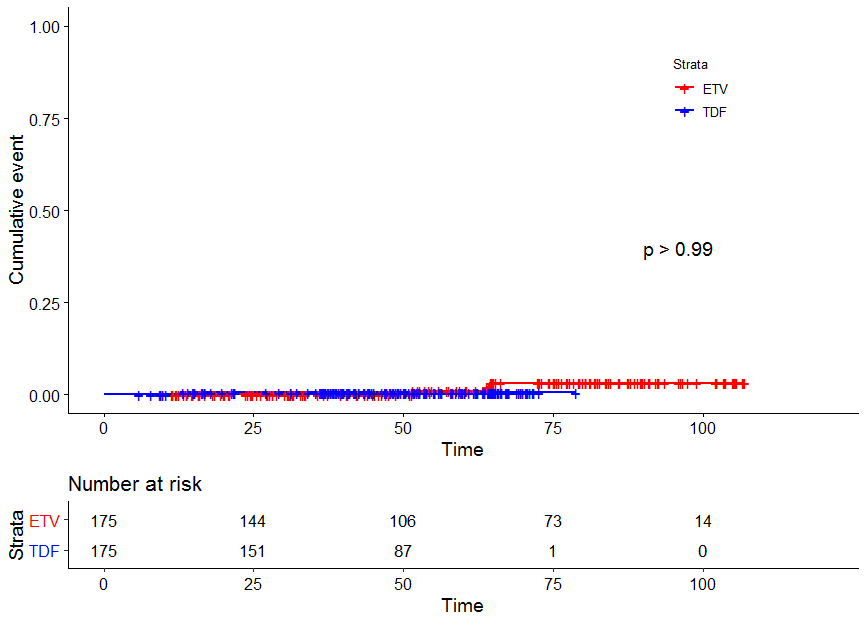
**

**Supplemental Content 6**. Baseline patient characteristics after inverse probability of treatment weighting

|  | Cohort for hepatocellular carcinoma | | | Cohort for death and transplantation | | | |  |
| --- | --- | --- | --- | --- | --- | --- | --- | --- |
| Characteristic | ETV  (n=168) | TDF  (n=213) | SMD | | ETV  (n=175) | TDF  (n=224) | SMD | |
| Age, mean ± SD, years | 45.2 ± 10.7 | 45.2 ± 11.3 | <0.001 | | 45.0 ± 10.7 | 45.1 ± 11.5 | <0.001 | |
| Male sex, n (%) | 93 (55.2) | 118 (55.3) | 0.001 | | 98 (55.8) | 125 (55.8) | <0.001 | |
| BMI, kg/m^2^, median (IQR) | 23.2  (21.2, 25.6) | 23.2  (21.0, 25.5) | 0.02 | | 23.1  (21.1, 25.6) | 23.2  (21.1, 25.5) | 0.04 | |
| Cirrhosis, n (%) | 59 (35.4) | 76 (35.6) | 0.004 | | 62 (35.3) | 79 (35.5) | 0.005 | |
| Diabetes, n (%) | 15 (8.8) | 17 (8.2) | 0.02 | | 16 (9.1) | 19 (8.7) | 0.02 | |
| Hepatitis e antigen positivity, n (%) | 100 (59.6) | 129 (60.4) | 0.02 | | 106 (60.4) | 136 (61.1) | 0.02 | |
| HBV DNA, log_10_(copies/mL), median (IQR) | 7.51  (6.70, 8.49) | 7.53  (6.43, 8.62) | <0.001 | | 7.62  (6.69, 8.52) | 7.54  (6.43, 8.65) | <0.001 | |
| Platelet, ×10^3^/μL, median (IQR) | 155  (112, 198) | 153  (111, 198) | 0.001 | | 158  (113, 199) | 154  (113, 200) | 0.002 | |
| Albumin, g/dL, median (IQR) | 4.1 (3.4, 4.5) | 4.2 (3.8, 4.5) | 0.004 | | 4.1 (3.7, 4.5) | 4.2 (3.8, 4.4) | 0.007 | |
| Bilirubin, mg/dL, median (IQR) | 0.76  (0.52, 1.23) | 0.80  (0.57, 1.12) | 0.06 | | 0.73  (0.51, 1.23) | 0.80  (0.56, 1.12) | 0.05 | |
| PT, INR, median (IQR) | 1.05  (1.00, 1.17) | 1.06  (1.00, 1.14) | 0.02 | | 1.05  (1.00, 1.17) | 1.06  (1.00, 1.14) | 0.02 | |
| ALT, IU/L, median (IQR) | 84.0  (36.8, 154.0) | 84.0  (42.0, 156.6) | 0.003 | | 85.7  (38.8, 154.2) | 84.0  (40.5, 156.3) | 0.003 | |
| Creatinine, mg/dL, median (IQR) | 0.9 (0.8, 1.0) | 0.9 (0.8, 1.0) | 0.002 | | 0.9 (0.8, 1.0) | 0.9 (0.8, 1.0) | 0.001 | |
| Child-Pugh score, median (IQR) | 5.0 (5.0, 6.0) | 5.0 (5.0, 5.0) | 0.01 | | 5.0 (5.0, 5.2) | 5.0 (5.0, 5.0) | 0.009 | |
| GAG-HCC score, median (IQR) | 85.2  (69.6, 103.8) | 84.5  (68.9, 106.8) | 0.004 | | 85.2  (70.4, 103.5) | 84.2  (68.8, 106.9) | 0.004 | |
| CU-HCC score, median (IQR) | 5.5  (4.0, 22.0) | 6.2  (4.0, 22.0) | 0.004 | | 5.5  (4.0, 22.0) | 7.0  (4.0, 22.0) | 0.004 | |
| PAGE-B score, median (IQR) | 12.0  (10.0, 16.0) | 12.0  (10.0, 17.0) | 0.004 | | 12.0  (9.5, 16.0) | 12.0  (9.4, 16.0) | 0.005 | |
| Treatment response at 1 year, n (%) |  |  |  | |  |  |  | |
| Biochemical | 109 (67.6) | 129 (61.4) | 0.13 | | 115 (68.9) | 130 (61.6) | 0.15 | |
| Serological* | 69 (45.0) | 98 (53.6) | 0.17 | | 70 (44.9) | 100 (63.6) | 0.18 | |
| Virological† | 116 (71.1) | 163 (77.2) | 0.14 | | 118 (71.0) | 164 (77.1) | 0.14 | |
| Sustained virological suppression, n (%) | 76 (46.8) | 123 (58.9) | 0.24 | | 72 (43.6) | 116 (54.7) | 0.22 | |
| Duration of follow-up, month, median (IQR) | 65.0  (32.2, 84.4) | 49.6  (38.5, 62.2) | 0.54 | | 64.3  (30.9, 84.2) | 49.0  (37.4, 61.9) | 0.52 | |

* Hepatitis e antigen seroconversion.

† Undetectable HBV DNA by polymerase chain reaction with a detection limit of 10 IU/mL.

ETV, entecavir; TDF, tenofovir disoproxil fumarate; SMD, standardized mean difference; SD, standard deviation; IQR, interquartile ranges; BMI, body mass index; HBV, hepatitis B virus; PT, prothrombin time; INR, international normalized ratio; ALT, alanine aminotransferase; GAG, guide with age, gender, HBV DNA, core promoter mutations and cirrhosis; CU, Chinese University.

**Supplemental Content 7 (a)**

**
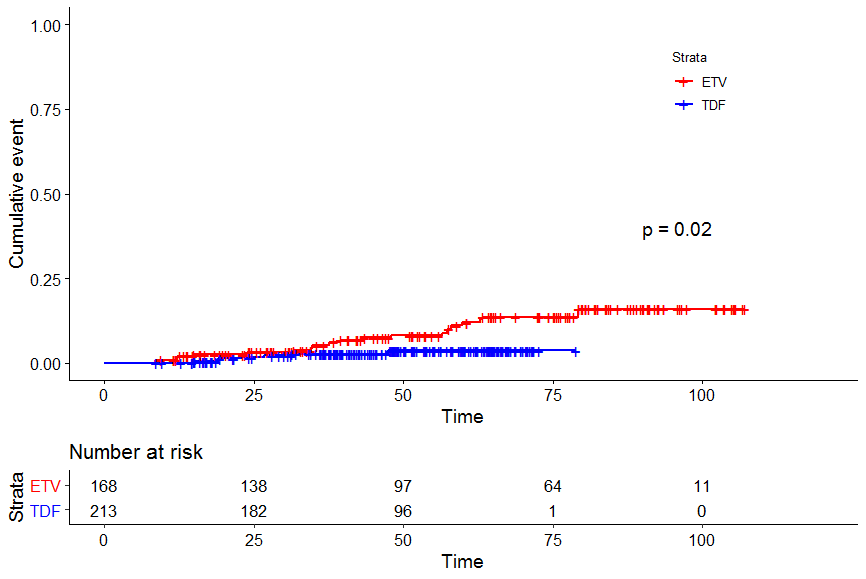
**

**Supplemental Content 7 (b)**

**
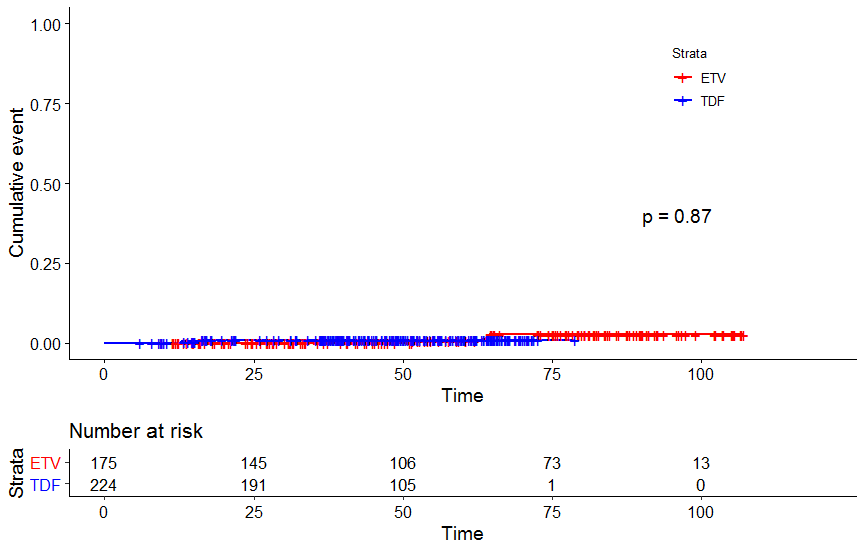
**

**Supplemental Content 8**. Baseline characteristics of subgroup of patients with cirrhosis

|  | Entire cirrhotic subgroup | | | Propensity score-matched cirrhotic subgroup | | | | | |  |
| --- | --- | --- | --- | --- | --- | --- | --- | --- | --- | --- |
|  |  |  |  | Hepatocellular carcinoma | | | Death and transplantation | | |  |
| Characteristic | ETV  (n=67) | TDF  (n=78) | *P* | ETV  (n=57) | TDF  (n=57) | SMD | ETV  (n=60) | TDF  (n=60) | SMD | |
| Age, mean ± SD, years | 49.5 ± 9.8 | 49.6 ± 9.5 | 0.61 | 50.2 ± 9.6 | 49.2 ± 9.4 | 0.11 | 50.2 ± 9.9 | 48.9 ± 10.1 | 0.13 | |
| Male sex, n (%) | 38 (56.7) | 48 (61.5) | 0.61 | 32 (56.1) | 35 (61.4) | 0.11 | 33 (55.0) | 35 (58.3) | 0.07 | |
| BMI, kg/m^2^, median (IQR) | 23.5 (21.7, 25.6) | 23.5 (21.3, 25.9) | 0.90 | 23.4 (21.7, 25.6) | 23.6 (21.3, 26.7) | 0.10 | 23.3 (21.7, 25.6) | 23.3 (21.0, 26.2) | 0.14 | |
| Diabetes, n (%) | 16 (23.9) | 10 (12.8) | 0.13 | 13 (22.8) | 10 (17.5) | 0.13 | 14 (23.3) | 7 (11.7) | 0.31 | |
| Hepatitis e antigen positivity, n (%) | 40 (63.5) | 40 (51.3) | 0.17 | 35 (61.4) | 33 (57.9) | 0.07 | 38 (63.3) | 33 (55.0) | 0.17 | |
| HBV DNA, log_10_(copies/mL), median (IQR) | 7.40 (6.57, 8.00) | 6.84 (6.12, 7.76) | 0.09 | 7.43 (6.74, 8.00) | 7.01 (6.34, 7.76) | 0.26 | 7.41 (6.63, 7.95) | 6.91 (6.23, 7.75) | 0.22 | |
| Platelet, x10^3^/μL, median (IQR) | 117  (79, 157) | 104  (77, 139) | 0.18 | 123  (80, 154) | 107  (77, 143) | 0.21 | 120  (80, 156) | 106  (77, 140) | 0.23 | |
| Albumin, g/dL, median (IQR) | 3.9  (3.1, 4.3) | 4.0  (3.2, 4.3) | 0.65 | 3.9 (3.2, 4.3) | 3.9 (3.1, 4.2) | 0.01 | 3.9 (3.2, 4.3) | 4.0 (3.3, 4.3) | 0.03 | |
| Bilirubin, mg/dL, median (IQR) | 1.03 (0.58, 1.50) | 0.92 (0.67, 1.40) | 0.94 | 1.11 (0.64, 1.45) | 0.97 (0.68, 1.40) | 0.07 | 1.03 (0.64, 1.44) | 0.90 (0.66, 1.29) | 0.08 | |
| PT, INR, median (IQR) | 1.17 (1.06, 1.40) | 1.11 (1.05, 1.24) | 0.17 | 1.17 (1.07, 1.38) | 1.11 (1.05, 1.25) | 0.16 | 1.17 (1.06, 1.34) | 1.11 (1.05, 1.24) | 0.17 | |
| ALT, IU/L, median (IQR) | 46.0 (30.0, 84.0) | 44.5 (26.8, 105.8) | 0.58 | 46.0 (30.0, 90.0) | 42.0 (26.0, 87.0) | 0.10 | 46.0 (30.8, 85.5) | 42.0 (25.8, 84.3) | 0.01 | |
| Creatinine, mg/dL, median (IQR) | 0.9  (0.8, 1.0) | 0.9  (0.8, 1.0) | 0.86 | 0.9  (0.8, 1.0) | 0.9  (0.8, 1.0) | 0.10 | 0.9  (0.8, 1.0) | 0.9  (0.8, 1.0) | 0.08 | |
| Child–Pugh score, median (IQR) | 5.0  (5.0, 7.0) | 5.0  (5.0, 6.0) | 0.11 | 5.5  (5.0, 7.0) | 5.0  (5.0, 6.0) | 0.15 | 5.5  (5.0, 7.0) | 5.0  (5.0, 6.0) | 0.18 | |
| GAG-HCC score, median (IQR) | 112.3 (103.5, 121.5) | 111.8 (104.6,  121.9) | 0.80 | 113.2 (103.7, 122.4) | 111.7  (104.7, 121.9) | 0.08 | 113.0 (103.5, 121.7) | 110.0  (103.9, 120.5) | 0.12 | |
| CU-HCC score, median (IQR) | 23.5 (22.0, 42.0) | 22.0 (22.0, 42.0) | 0.07 | 23.5 (19.0, 40.5) | 22.0 (19.0, 40.5) | 0.06 | 23.5 (19.0, 39.4) | 22.0 (19.0, 39.0) | 0.14 | |
| PAGE-B score, median (IQR) | 15.0 (12.0, 18.0) | 16.0 (13.0, 19.0) | 0.22 | 15.0 (12.0, 18.0) | 16.0 (13.0, 18.0) | 0.18 | 15.0 (12.0, 18.0) | 16.0 (13.0, 18.0) | 0.14 | |
| Treatment response at 1 year, n (%) |  |  |  |  |  |  |  |  |  | |
| Biochemical | 34 (52.3) | 36 (48.0) | 0.74 | 27 (48.2) | 27 (48.2) | <0.001 | 29 (49.2) | 29 (48.3) | 0.02 | |
| Serological* | 26 (44.1) | 30 (50.0) | 0.58 | 24 (47.1) | 24 (52.2) | 0.10 | 26 (48.2) | 25 (52.1) | 0.08 | |
| Virological† | 49 (76.6) | 60 (76.9) | >0.99 | 42 (73.7) | 43 (75.4) | 0.04 | 44 (73.3) | 46 (76.7) | 0.08 | |
| Sustained virological suppression, n (%) | 28 (41.8) | 41 (52.6) | 0.24 | 27 (47.4) | 31 (54.4) | 0.14 | 25 (41.7) | 29 (48.3) | 0.13 | |
| Duration of follow-up, month, median (IQR) | 74.4 (28.9, 85.9) | 49.2 (36.4, 62.6) | 0.003 | 76.0 (37.5, 86.2) | 52.3 (37.7, 62.4) | 0.72 | 75.2 (35.3, 86.0) | 52.1 (37.7, 63.3) | 0.60 | |

* Hepatitis e antigen seroconversion.

† Undetectable HBV DNA by polymerase chain reaction with a detection limit of 10 IU/mL.

ETV, entecavir; TDF, tenofovir disoproxil fumarate; SMD, standardised mean difference; SD, standard deviation; IQR, interquartile ranges; BMI, body mass index; HBV, hepatitis B virus; PT, prothrombin time; INR, international normalized ratio; ALT, alanine aminotransferase; GAG, guide with age, gender, HBV DNA, core promoter mutations and cirrhosis; CU, Chinese University.

**Supplemental Content 9**. Risk of hepatocellular carcinoma or death and transplantation in a subgroup of patients with cirrhosis on univariate analysis

| Variable | Risk of HCC |  | Risk of death and transplantation |  |
| --- | --- | --- | --- | --- |
|  | Hazard ratio (95% CI) | *P* | Hazard ratio (95% CI) | *P* |
| TDF treatment | 0.30 (0.11‒0.84) | 0.021 | 0.58 (0.10‒3.25) | 0.53 |
| Age, per 1 year | 1.03 (0.98‒1.08) | 0.27 | 0.98 (0.90‒1.07) | 0.64 |
| Male sex | 1.41 (0.58‒3.41) | 0.45 | 57.68 (0.08‒INF) | 0.23 |
| BMI, per 1 kg/m^2^ | 1.18 (0.98‒1.43) | 0.09 | 1.27 (0.96‒1.67) | 0.09 |
| Diabetes | 4.13 (1.74‒9.83) | 0.001 | 2.03 (0.37‒11.10) | 0.42 |
| Hepatitis e antigen | 0.77 (0.33‒1.82) | 0.55 | 1.05 (0.18‒6.31) | 0.95 |
| HBV DNA, per 1 log_10_(copies/mL) | 1.30 (0.85‒1.98) | 0.22 | 1.20 (0.57‒2.54) | 0.63 |
| Platelet, per 1000/μL | 1.00 (0.99‒1.01) | 0.46 | 0.99 (0.97‒1.01) | 0.23 |
| Albumin, per 1 g/dL | 0.74 (0.40‒1.35) | 0.32 | 0.23 (0.08‒0.68) | 0.007 |
| Bilirubin, per 1 mg/dL | 1.16 (0.82‒1.63) | 0.41 | 1.40 (0.95‒2.08) | 0.09 |
| PT, per 1 INR | 1.19 (0.27‒5.20) | 0.82 | 2.89 (0.57‒14.69) | 0.20 |
| ALT, per 1 IU/L | 1.00 (1.00‒1.00) | 0.79 | 0.98 (0.94‒1.01) | 0.15 |
| Creatinine, per 1 mg/dL | 1.29 (0.20‒8.48) | 0.77 | 4.14 (0.90‒18.96) | 0.07 |
| Child-Pugh score, per 1 point | 1.08 (0.84‒1.38) | 0.57 | 1.58 (1.16‒2.15) | 0.003 |
| Virological response* | 0.67 (0.26‒1.74) | 0.41 | 0.57 (0.10‒3.10) | 0.51 |
| Sustained virological suppression | 1.35 (0.57‒3.22) | 0.49 | 0.29 (0.03‒2.55) | 0.26 |

* Undetectable HBV DNA by polymerase chain reaction with a detection limit of 10 IU/mL.

HCC, hepatocellular carcinoma; CI, confidence interval; TDF, tenofovir disoproxil fumarate; BMI, body mass index; HBV, hepatitis B virus; PT, prothrombin time; INR, international normalized ratio; ALT, alanine aminotransferase.

**Supplemental Content 10**. Supplemental Content 10. Baseline characteristics of subgroup of elderly patients (≥ 50 years)

|  | Entire elderly subgroup | | | Propensity score-matched elderly subgroup | | | | | |
| --- | --- | --- | --- | --- | --- | --- | --- | --- | --- |
|  |  |  |  | Hepatocellular carcinoma | | | Death and transplantation | | |
| Characteristic | ETV  (n=61) | TDF  (n=82) | *P* | ETV  (n=58) | TDF  (n=58) | SMD | ETV  (n=60) | TDF  (n=60) | SMD |
| Age, mean ± SD, years | 57.2 ± 6.5 | 56.6 ± 5.1 | 0.96 | 57.2 ± 6.4 | 57.3 ± 5.0 | 0.02 | 57.3 ± 6.5 | 56.9 ± 5.0 | 0.07 |
| Male sex, n (%) | 30 (49.2) | 46 (56.1) | 0.50 | 29 (50.0) | 33 (56.9) | 0.14 | 29 (48.3) | 33 (55.0) | 0.13 |
| BMI, kg/m^2^, median (IQR) | 22.6 (21.4, 25.7) | 23.2 (21.5, 25.5) | 0.66 | 22.6 (21.4, 25.6) | 23.2 (21.5, 24.8) | 0.09 | 22.6 (21.5, 25.6) | 23.5 (21.7, 25.3) | 0.04 |
| Cirrhosis, n (%) | 34 (55.7) | 43 (52.4) | 0.74 | 31 (53.5) | 30 (51.7) | 0.03 | 33 (55.0) | 31 (51.7) | 0.07 |
| Diabetes, n (%) | 12 (19.7) | 11 (13.4) | 0.36 | 10 (17.2) | 7 (12.1) | 0.15 | 11 (18.3) | 8 (13.3) | 0.14 |
| Hepatitis e antigen positivity, n (%) | 34 (56.7) | 42 (51.2) | 0.61 | 32 (55.2) | 31 (53.5) | 0.03 | 34 (56.7) | 32 (53.3) | 0.07 |
| HBV DNA, log_10_(copies/mL), median (IQR) | 7.43 (6.44, 8.44) | 7.15 (6.25, 8.42) | 0.95 | 7.43 (6.58, 8.46) | 7.10 (6.34, 8.14) | 0.09 | 7.42 (6.46, 8.42) | 7.10 (6.32, 8.23) | 0.03 |
| Platelet, ×10^3^/μL, median (IQR) | 128  (88, 172) | 131  (96, 170) | 0.91 | 130  (93, 176) | 138  (95, 179) | 0.09 | 130  (90, 179) | 130  (102, 167) | 0.13 |
| Albumin, g/dL, median (IQR) | 4.0  (3.3, 4.3) | 4.1  (3.6, 4.3) | 0.47 | 4.0  (3.3, 4.3) | 4.1  (3.8, 4.3) | 0.14 | 4.0  (3.3, 4.3) | 4.1  (3.7, 4.3) | 0.07 |
| Bilirubin, mg/dL, median (IQR) | 1.11 (0.62, 1.56) | 0.81 (0.56, 1.29) | 0.13 | 1.12 (0.64, 1.55) | 0.84 (0.62, 1.28) | 0.28 | 1.11 (0.64, 1.53) | 0.90 (0.60, 1.29) | 0.27 |
| PT, INR, median (IQR) | 1.08 (1.03, 1.24) | 1.09 (1.02, 1.18) | 0.45 | 1.09 (1.03, 1.24) | 1.09 (1.01, 1.16) | 0.18 | 1.08 (1.03, 1.24) | 1.10 (1.03, 1.16) | 0.15 |
| ALT, IU/L, median (IQR) | 80.0 (33.0, 142.5) | 51.5 (27.8, 105.3) | 0.29 | 83.5 (34.5, 142.8) | 51.5 (29.0, 105.5) | 0.07 | 81.5 (33.8, 142.3) | 52.0 (33.5, 104.5) | 0.11 |
| Creatinine, mg/dL, median (IQR) | 0.9  (0.8, 1.1) | 0.9  (0.8, 1.0) | 0.85 | 0.9  (0.8, 1.0) | 0.9  (0.8, 1.1) | 0.06 | 0.9  (0.8, 1.0) | 0.9  (0.8, 1.1) | 0.06 |
| Child–Pugh score, median (IQR) | 5.0  (5.0, 7.0) | 5.0  (5.0, 6.0) | 0.14 | 5.0  (5.0, 6.0) | 5.0  (5.0, 5.0) | 0.16 | 5.0  (5.0, 6.0) | 5.0  (5.0, 5.3) | 0.16 |
| GAG-HCC score, median (IQR) | 106.9 (90.6, 121.7) | 105.8 (88.0, 120.9) | 0.77 | 105.2 (90.5, 121.2) | 104.6 (90.7, 120.7) | 0.02 | 105.2 (90.8, 121.0) | 105.8 (89.6, 120.9) | 0.03 |
| CU-HCC score, median (IQR) | 22.0 (7.0, 33.8) | 20.5 (7.0, 25.3) | 0.41 | 22.0 (7.0, 28.5) | 19.0 (7.0, 23.5) | 0.15 | 22.0 (7.0, 28.5) | 19.8 (7.0, 23.5) | 0.09 |
| PAGE-B score, median (IQR) | 15.0 (12.0, 20.0) | 17.5 (12.0, 18.5) | 0.58 | 15.0 (12.0, 19.5) | 17.5 (12.0, 18.0) | 0.17 | 15.0 (12.0, 18.5) | 17.0 (12.0, 18.0) | 0.14 |
| Treatment response at 1 year, n (%) |  |  |  |  |  |  |  |  |  |
| Biochemical | 35 (58.3) | 38 (49.4) | 0.31 | 33 (57.9) | 26 (44.8) | 0.26 | 34 (57.6) | 31 (51.7) | 0.12 |
| Serological* | 21 (38.9) | 31 (48.4) | 0.35 | 20 (39.2) | 24 (50.0) | 0.22 | 21 (39.6) | 26 (52.0) | 0.25 |
| Virological† | 46 (75.4) | 60 (75.9) | >0.99 | 43 (74.1) | 44 (75.9) | 0.04 | 45 (75.0) | 46 (76.7) | 0.04 |
| Sustained virological suppression, n (%) | 30 (49.1) | 49 (59.8) | 0.16 | 29 (50.0) | 33 (56.9) | 0.14 | 28 (46.7) | 33 (55.0) | 0.17 |
| Duration of follow-up, month, median (IQR) | 72.6 (39.1, 83.4) | 49.1 (36.9, 63.8) | <0.001 | 73.0 (44.4, 83.6) | 50.4 (38.1, 64.1) | 0.73 | 72.7 (40.8, 83.1) | 48.8 (37.6, 61.2) | 0.70 |

* Hepatitis e antigen seroconversion.

† Undetectable HBV DNA by polymerase chain reaction with a detection limit of 10 IU/mL.

ETV, entecavir; TDF, tenofovir disoproxil fumarate; SMD, standardised mean difference; SD, standard deviation; IQR, interquartile ranges; BMI, body mass index; HBV, hepatitis B virus; PT, prothrombin time; INR, international normalized ratio; ALT, alanine aminotransferase; GAG, guide with age, gender, HBV DNA, core promoter mutations and cirrhosis; CU, Chinese University

**Supplemental Content 11**. Risk of hepatocellular carcinoma or death and transplantation in a subcohort of elderly patients on univariate analysis

| Variable | Risk of HCC |  | Risk of death and transplantation |  |
| --- | --- | --- | --- | --- |
|  | Hazard ratio (95% CI) | *P* | Hazard ratio (95% CI) | *P* |
| TDF treatment | 0.41 (0.13‒1.35) | 0.14 | 1.54 (0.14‒17.01) | 0.72 |
| Age, per 1 year | 1.06 (0.97‒1.15) | 0.19 | 0.90 (0.69‒1.17) | 0.43 |
| Male sex | 1.42 (0.49‒4.11) | 0.52 | 64.72 (0.01‒INF) | 0.38 |
| BMI, per 1 kg/m^2^ | 1.28 (0.97‒1.68) | 0.08 | 2.05 (0.62‒6.84) | 0.24 |
| Diabetes | 9.64 (3.34‒27.85) | <0.001 | 2.80 (0.25‒30.93) | 0.40 |
| Hepatitis e antigen | 0.69 (0.24‒1.99) | 0.49 | 0.01 (0.00‒1186.49) | 0.46 |
| HBV DNA, per 1 log_10_(copies/mL) | 1.13 (0.74‒1.74) | 0.57 | 0.74 (0.31‒1.80) | 0.51 |
| Platelet, per 1000/μL | 1.00 (0.98‒1.01) | 0.33 | 0.98 (0.95‒1.01) | 0.14 |
| Albumin, per 1 g/dL | 0.73 (0.34‒1.55) | 0.41 | 0.20 (0.05‒0.91) | 0.037 |
| Bilirubin, per 1 mg/dL | 1.20 (0.78‒1.84) | 0.40 | 0.76 (0.15‒4.01) | 0.75 |
| PT, per 1 INR | 1.04 (0.13‒8.24) | 0.97 | 3.77 (0.34‒41.73) | 0.28 |
| ALT, per 1 IU/L | 1.00 (0.99‒1.00) | 0.38 | 0.93 (0.85‒1.03) | 0.15 |
| Creatinine, per 1 mg/dL | 0.82 (0.15‒4.40) | 0.82 | 2.12 (0.82‒5.50) | 0.12 |
| Child–Pugh score, per 1 point | 1.14 (0.85‒1.53) | 0.38 | 1.63 (1.05‒2.52) | 0.028 |
| Virological response* | 0.67 (0.21‒2.14) | 0.49 | 30.89 (0.00‒INF) | 0.55 |
| Sustained virological suppression | 1.17 (0.40‒3.41) | 0.77 | 0.89 (0.06‒14.23) | 0.93 |

* Undetectable HBV DNA by polymerase chain reaction with a detection limit of 10 IU/mL.

HCC, hepatocellular carcinoma; CI, confidence interval; TDF, tenofovir disoproxil fumarate; BMI, body mass index; HBV, hepatitis B virus; PT, prothrombin time; INR, international normalized ratio; ALT, alanine aminotransferase.
